# Effects of systemic pretreatment with the NAALADase inhibitor 2-PMPA on oral methamphetamine reinforcement in C57BL/6J mice

**DOI:** 10.3389/fpsyt.2024.1297275

**Published:** 2024-04-04

**Authors:** Elissa K. Fultz, Andrea Y. T. Nei, Joyce C. Chi, Jacqueline N. Lichter, Karen K. Szumlinski

**Affiliations:** ^1^ Department of Psychological and Brain Sciences, University of California, Santa Barbara, Santa Barbara, CA, United States; ^2^ Department of Molecular, Cellular and Developmental Biology, University of California, Santa Barbara, Santa Barbara, CA, United States; ^3^ Neuroscience Research Institute, University of California, Santa Barbara, Santa Barbara, CA, United States

**Keywords:** operant-conditioning, NAAG, 2-PMPA, mouse model, methamphetamine methamphetamine, NAALADase inhibitor, reinforcement

## Abstract

**Introduction:**

Repeated exposure to methamphetamine (MA) in laboratory rodents induces a sensitization of glutamate release within the corticoaccumbens pathway that drives both the rewarding and reinforcing properties of this highly addictive drug. Such findings argue the potential for pharmaceutical agents inhibiting glutamate release or its postsynaptic actions at glutamate receptors as treatment strategies for MA use disorder. One compound that may accomplish both of these pharmacological actions is the *N*-acetylated-alpha-linked-acidic dipeptidase (NAALADase) inhibitor 2-(phosphonomethyl)pentanedioic acid (2-PMPA). 2-PMPA elevates brain levels of the endogenous agonist of glutamate mGluR3 autoreceptors, *N*-acetyl-aspartatylglutamate (NAAG), while potentially acting as an NMDA glutamate receptor antagonist. Of relevance to treating psychomotor stimulant use disorders, 2-PMPA is reported to reduce indices of both cocaine and synthetic cathinone reward, as well as cocaine reinforcement in preclinical rodent studies.

**Method:**

Herein, we conducted three experiments to pilot the effects of systemic pretreatment with 2-PMPA (0-100 mg/kg, IP) on oral MA self-administration in C57BL/6J mice. The first experiment employed female mice with a prolonged history of MA exposure, while the mice in the second (females) and third (males and females) experiment were MA-naïve prior to study. In all experiments, mice were trained daily to nose-poke for delivery of unadulterated MA solutions until responding stabilized. Then, mice were pretreated with 2-PMPA prior to operant-conditioning sessions in which nose-poking behavior was reinforced by delivery of 120 mg/L or 200 mg/L MA (respectively, in Experiments 1 and 2/3).

**Results:**

Contrary to our expectations, 30 mg/kg 2-PMPA pretreatment altered neither appetitive nor consummatory measures related to MA self-administration. In Experiment 3, 100 mg/kg 2-PMPA reduced responding in the MA-reinforced hole, as well as the number of reinforcers earned, but did not significantly lower drug intake.

**Discussion:**

These results provide mixed evidenced related to the efficacy of this NAALADase inhibitor for reducing oral MA reinforcement in female mice.

## 1 Introduction

According to the 2023 World Drug Report (1), the global prevalence of amphetamine-type stimulant (ATS) use [including that of methamphetamine (MA)], has increased significantly over the past decade, with approximately 36 million people (or 0.7% of the world’s population) reporting ATS use in 2021. Indicative of a closing of the gender gap in ATS use, 45% of individuals reporting current ATS use are women, but only 1 in 4 women receive treatment (1). This is very concerning as it is generally accepted that women progress along the addiction landscape faster than men (1–3). Further, women are reported to: start experimenting with MA at an earlier age than men (4), be more likely to experience positive moods in response to psychomotor stimulant drugs (5), exhibit lower drug abstinence rates (6) and experience comorbid psychiatric symptoms (7). The preclinical rodent literature regarding sex differences in MA reward and reinforcement aligns with clinical findings with most studies reporting a higher propensity of female rodents to acquire self-administration behavior, respond for, and consume MA when the drug is available intravenously (8–12) or orally [ (13); but see (14, 15)]. Female rodents also exhibit greater reactivity to MA-associated interoceptive and exteroceptive cues following drug abstinence than males (16–18). Further, unlike males (19), female C57BL/6J (B6) mice readily acquire oral MA self-administration even when their initial perception of the drug effect is aversive (20). Such preclinical findings highlight the importance of not only understanding how biological sex modulates vulnerability to and the progression of MA use disorder, but also determining the efficacy of potential pharmacotherapies in female subjects.

Over the past decade, accumulating preclinical evidence supports both correlative and causal links between potentiated indices of glutamate signaling within corticoaccumbens pathways and MA use disorder-related behavior, including: self-administration, MA-induced reinstatement of drug-seeking after abstinence or extinction, incubation of MA-craving, and conditioned place-preference (19, 21–29). For example, subchronic exposure to subtoxic MA doses (≤ 2 mg/kg) elevates extracellular glutamate within the nucleus accumbens (19, 30) and induces a sensitization of drug-induced glutamate release both the nucleus accumbens (19, 23, 30) and prefrontal cortex (23, 31). Additionally, repeated exposure to subtoxic MA doses alter the expression of both pre- and post-synaptic glutamate receptors in both brain regions (19, 22, 23, 32). Further, NMDA glutamate receptor antagonists attenuate MA-conditioned reward and behavioral sensitization (26), negative allosteric modulators of mGlu5 and agonists at mGlu2/3 glutamate receptors reduce intravenous MA self-administration (32, 33), as well as the reinstatement (32–34) and incubation (35) of MA-seeking in rats, while pharmacological manipulations of extracellular glutamate in the NAC bidirectionally regulate the expression of MA-conditioned reward in mice (19). Taken together, the results of the studies to date point to blunting MA-induced glutamate release and/or its ability to stimulate excitatory postsynaptic receptors as potential strategies to curb MA use disorder-related behavior, including drug-taking.

In this regard, the *N*-acetylated-alpha-linked-acidic dipeptidase (NAALADase) inhibitor 2-(phosphonomethyl)pentanedioic acid (2-PMPA) (36) may be a promising potential pharmacotherapy for treating MA use disorder. *N*-acetyl-aspartylglutamate (NAAG) is the most abundant neuropeptide in the mammalian brain and has been identified as both an endogenous mGlu3 glutamate receptor agonist (37), as well as an NMDA glutamate receptor antagonist (38). NAAG can functionally prevent excessive glutamate release via stimulation of presynaptic mGlu3 autoreceptors (39, 40), while simultaneously preventing NMDA-dependent depolarization of postsynaptic neurons (41). Of clinical relevance, magnetic resonance spectroscopy studies indicate lower NAAG levels within the dorsolateral prefrontal cortex, as well as other cortical areas, of humans with methamphetamine use disorder [e.g., (42, 43)] NAAG is inactivated by NAALADase and pharmacological inhibition of NAALADase activity by 2-PMPA or other inhibitors elevates brain NAAG levels [e.g., (44, 45).], which augments mGlu3-dependent inhibition of glutamate release (46) that is necessary for certain addiction-related behaviors (32, 46, 47). Supporting the potential “anti-addiction” efficacy of 2-PMPA for treating stimulant use disorders, systemic administration inhibits cocaine-kindled seizures (48), cocaine- induced behavioral sensitization (49), place-preference (50), and reinstatement of cocaine-seeking (46), as well as intravenous (**IV**) cocaine self-administration (46, 51). Systemic 2-PMPA pretreatment is also reported to blunt the locomotor-stimulating properties of amphetamine (52) and effectively block a place-preference induced by the synthetic cathinone 3,4-methylenedioxypyrovalerone (MDPV) (53). Even though stimulating mGlu3 receptors with exogenous agonists can reduce the expression of behaviors in rodent models of MA use disorder (c.f., 34), to the best of our knowledge, no study has examined for the effects of 2-PMPA on MA reward or reinforcement.

Thus, the goal of this study was to examine the effects of 2-PMPA at doses demonstrated to be effective at attenuating stimulant-induced changes in behavior in male rats or mice [e.g., 3-100 mg/kg; (46, 48–53)] on active MA self-administration. For this, we employed a procedurally facile operant-conditioning paradigm in which C57BL/6J (B6) mice are trained daily (1 h/day) to nose-poke for delivery of unadulterated solutions of MA (13, 19, 20, 54, 55). As female B6 mice consume more MA than males under our operant-conditioning procedures (13), our study commenced with two experiments conducted in female mice to provide a high baseline of MA self-administration upon which to gauge the potential “anti-addictive” effects of 2-PMPA (3 and 30 mg/kg). As the results of these first two studies were negative, a third experiment was conducted that included both males and females, as well as a higher 2-PMPA dose [100 mg/kg; (48–52)].

## 2 Methods

### 2.1 Subjects

Inbred, female and male, C57BL/6J (B6) mice (Jackson Laboratories, Sacramento, CA; 10-12 weeks of age) were housed in groups of four within polycarbonate cages under standard conditions. The mice were housed under a reverse 12-hour light/dark cycle (lights off: 11:00) for a minimum of 7 days prior to the start of operant-conditioning procedures. Food and water were available *ad libitum* with the exception of the time animals were engaged in behavioral testing. The mice employed in Experiment 1 were derived from an earlier study of the relationship between the motivational valence of MA as determined by conditioned place-preference (4 injections of 2 mg/kg MA) and subsequent MA self-administration (20) and had a relatively lengthy MA self-administration history prior to 2-PMPA testing (see Sect. 2.3 below). The mice employed in Experiments 2 and 3 were MA-naïve at the start of the study. The experiments followed a protocol consistent with NIH guidelines presented in the recently revised *Guide for Care and Use of Laboratory Animals* (NIH publication No. 80-23, revised 2014) and approved by the IACUC of the University of California, Santa Barbara under protocol 829.3 (Experiments 1 and 2) and 829.4 (Experiment 3).

### 2.2 Operant-conditioning for oral MA reinforcement

The procedures to induce operant-conditioning for an oral MA reinforcer were similar to those described in prior reports (13, 19, 20, 54, 55). Operant-conditioning involved daily 1-h training sessions in which mice were required to nose-poke for delivery of unadulterated solutions of MA (prepared in tap water; reinforcer volume=20 µl) under a fixed ratio 1 (FR1) schedule of reinforcement. Operant-conditioning occurred in standard mouse operant chambers (MedAssociates, St Albans, VT), fitted with 2 nose-poke holes and a liquid receptacle located between the holes, all housed within ventilated sound-attenuated chambers. For all operant-conditioning sessions, responses in the active (MA-associated) hole resulted in the activation of the infusion pump, delivery of 20 µl of the MA reinforcer into the receptacle, and the presentation of a 20-sec light/tone compound stimulus. During the 20-sec MA-delivery period, further responding in the active hole was recorded but had no programmed consequences. Throughout the session, responding in the inactive hole had no programmed consequences but was recorded to index the selectivity of responding and general motor activity. At the end of each 1-h operant session, the volume of solution remaining in the receptacle was determined by pipetting [e.g., (13, 19, 20)] and mice were returned to the colony room and left undisturbed until the next day. Total MA intake was determined each day by subtracting the volume of MA remaining in the receptacle from the total volume delivered and was expressed as a function of body weight (in mg/kg), which was determined weekly during training and prior to each 2-PMPA testing session as described below.

### 2.3 Experiment 1: effects of 2-PMPA in female mice with an extensive MA history

To pilot a potential effect of 2-PMPA pretreatment on oral MA self-administration, Experiment 1 employed female mice from an earlier study that were well-trained in MA self-administration. As detailed in Shab et al. (20), the mice in Experiment 1 first underwent MA place-conditioning procedures during which they received 4 intraperitoneal (IP) injections of 2 mg/kg. Following testing for their place-preference, mice then underwent operant-conditioning procedures during which they were initially trained to respond for 20 mg/L MA, and then underwent demand-response testing at this concentration. Following establishment of the demand-response function, the concentration of the MA reinforcer was progressively increased until mice were reliably responding for 400 mg/L MA. Transition to the next MA concentration required that the mice earn a minimum of 10 reinforcers during a 1-h session, with greater than 70% of their responding directed towards the active lever for 2-3 consecutive days. Following completion of dose-response testing, the MA reinforcer was lowered to 120 mg/L – a concentration that lies on the ascending limb of the dose-response function for responding in the active hole (13, 20, 55). We opted to lower the MA reinforcer dose from 400 to 120 mg/L MA based on the results of a prior intravenous cocaine study indicating that pretreatment with 10 or 30 mg/kg 2-PMPA is more effective at reducing the self-administration of lower, rather than higher, cocaine doses (46).

Once mice achieved stable responding for the 120 mg/L MA solution, testing for the effects of 2-PMPA pretreatment began. Experiment 1 employed a quasi-within-subjects design in which each mouse received an intraperitoneal injection of 2 of 3 possible 2-PMPA doses (0, 3 or 30 mg/kg IP; Tocris Biosciences, Minneapolis, MN, USA). While it would have been ideal to test each mouse under all 3 doses, the availability of the operant chambers was limited due to other large-scale on-going studies [e.g., (52, 53)]. A 50 mM 4-(2-hydroxyethyl)-1-piperazineethanesulfonic acid (HEPES) solution (in sterile water; vol=10 mg/L) was employed as the vehicle (46) and the maximum 2-PMPA dose of 30 mg/kg was selected as it is sufficient to robustly lower IV cocaine self-administration in rats and blunt the glutamate-elevating effects of this stimulant (46). The two 2-PMPA pretreatments were spaced 3 days apart to examine for any carry-over effects. The order of dosing was pseudo-randomized such that 1/3 of the mice received each of the three 2-PMPA doses on each test day and no animal received the same pretreatment twice. We also ensured that half of the mice received a lower dose followed by a higher dose and vice versa. Thirty minutes following the IP 2-PMPA injection, mice were placed into their assigned operant-chamber and self-administration behavior recorded for 1 h. Given that the experimental design did not permit a within-subjects analysis of all three 2-PMPA doses, dose was treated as a between-subjects factor and the data were analyzed using a one-way ANOVA, with alpha set at 0.05. Prior to conducting any statistical analyses, extreme outliers were examined using the ± 3 × IQR rule, which identified one mouse pretreated with 10 mg/kg that exhibited very high (186) responses in the inactive hole. Thus, this mouse was dropped from the analyses of both this variable and the variable of response allocation (i.e., the relative responding in active vs. inactive hole). IBM SPSS Statistics software (version 27.0 for PC) was used for all statistical tests, and GraphPad Prism software (version 9.3.1 for PC) was used to create all graphs.

### 2.4 Experiment 2: effects of 2-PMPA in female mice trained on 200 mg/L MA

To determine whether the failure to detect an effect of 2-PMPA on MA self-administration in Experiment 1 (see Results) might relate to the complicated prior MA history of the mice, we conducted a second pilot study in which MA-naïve female B6 mice were trained for 14 consecutive days to self-administer 200 mg/L MA prior to testing. At the time of Experiment 2, we knew that 200 mg/L MA lay on the ascending limb of the dose-response function for MA reinforcement in female B6 mice (20) but not whether this concentration would support the acquisition of self-administration behavior as B6 mice reportedly exhibit low oral MA consumption in the home cage and find higher MA doses aversive (15, 56). Having established the acquisition and stability of self-administration behavior (see Results), we then tested the effects of 2-PMPA (3, 10 and 30 mg/kg, IP; Tocris Biosciences) on responding for the 200 mg/L MA solution. As the mice in Experiment 2 had never received an IP injection, we opted to first habituate the mice to IP injections by pretreating all the mice with the 50 mM HEPES vehicle 30 min prior to an initial test session. A cursory analysis of responding indicated that the initial HEPES injection significantly reduced active hole-poking well below the average of their last 3 days prior to injection in 12 of the 31 mice tested in Experiment 2 and this low level of responding persisted in these mice across the next 5 days of self-administration (see Results). Thus, we decided to continue HEPES pretreatment in all of the mice in Experiment, every 2-3 days, for a total of 3 pretreatments to the hopes of habituating the “reactive” mice to the vehicle injection. As their responding did not return to their pre-HEPES baseline (see Results), we then considered the average behavior across the 3 HEPES injections as the new baseline upon which to compare the effects of 2-PMPA. Three days following their 3^rd^ HEPES injection, mice were assigned to receive one of three 2-PMPA (3, 10, 30 mg/kg), ensuring that the average responding under HEPES pretreatment was equivalent across the three 2-PMPA doses by one-way ANOVAs. We included the 10 mg/kg 2-PMPA dose in Experiment 2 as this dose is reported to also be effective at reducing IV cocaine self-administration in rats (46, 51) and a prior dose-response study of the effects of 2-PMPA pretreatment on alcohol consumption by P rats revealed an inverted U-shape dose-response function (57). As in Experiment 1, mice were injected with 2-PMPA 30 min prior to the 1-h MA self-administration test session.

As conducted for Experiment 1, prior to statistical analyses of each variable, extreme outliers were explored using the ± 3 × IQR rule but failed to identify any mice. Thus, 11 mice were pretreated with 3 mg/kg, and 10 mice were each pretreated with 10 or 30 mg/kg 2-PMPA. Given the experimental design, the data were analyzed using a mixed model ANOVA with Pretreatment as a within-subjects factor (average HEPES vs. PMPA) and dose (3, 10 and 30 mg/kg) as a between-subjects factor. The same statistical and graphing software were employed in Experiment 2 as those employed in Experiment 1.

### 2.5 Experiment 3: effects of 2-PMPA in male and female mice trained on 200 mg/L MA

To determine whether the lack of any 2-PMPA effect on MA self-administration in Experiments 1 and 2 reflected insufficient 2-PMPA dosing or the sex of the subjects, Experiment 3 was conducted in which male and female B6 mice were trained to respond for 200 mg/L MA until responding stabilized. Mice were then pretreated IP with either 0, 30 or 100 mg/kg 2-PMPA, with dosing order counterbalanced across mice in a within-subjects design. MA self-administration resumed following each pretreatment and continued until responding re-stabilized, which required 2-3 days for most mice. One female failed to meet the acquisition criteria for MA self-administration and thus, did not undergo 2-PMPA treatment. Following the completion of dose-response testing under MA reinforcement, the MA solution was substituted for a 20% (w/v) sucrose solution and the mice were allowed to self-administer sucrose until responding stabilized (2-4 days for all mice, including the female that failed to acquire MA self-administration). Upon the stabilization of responding for sucrose, the mice were then pretreated with either 0 or 100 mg/kg 2-PMPA (i.e., the highest 2-PMPA dose assayed under MA self-administration procedures), in a counterbalanced fashion across subjects within each sex, with 2-3 days allowed between pretreatments for re-stabilization of responding. For Experiment 3, 2-PMPA was obtained from MedSciExpress USA (Monmouth Junction, NJ, USA) and was soluble in water. Thus, water served as the vehicle for Experiment 3 (vol=10 ml/kg), which avoided the issues associated with the HEPES pretreatment detected in Experiment 2.

The data in Experiment 3 were analyzed using a Sex X Dose ANOVA, with repeated measures on the Dose factor (3 levels for MA self-administration; 2 levels for sucrose self-administration). Analysis for extreme outliers using the ± 3 × IQR rule failed to identify outliers in this experiment. Thus, the final samples sizes were n=12 for males for both MA and sucrose self-administration, n=11 for females during MA self-administration and n=12 for female during sucrose self-administration. IBM SPSS Statistics software (version 29.0 for PC) was used for all statistical tests for Experiment 3, and GraphPad Prism software (version 10.1.2 for PC) was used to create all graphs.

## 3 Results

### 3.1 2-PMPA effects on operant-responding for 120 mg/L MA in MA-experienced female B6 mice

The results pertaining to the acquisition of operant-conditioning for the 20 mg/L MA solution, as well as the dose- and demand-response functions for oral MA self-administration were published in Shab et al. (20). As summarized in **Table 1A**, no group differences were detected for the average number of responses in the active or inactive hole and no difference was detected for the allocation of total responding towards the active hole (response allocation) prior to each of the two pretreatment days. Likewise, no group differences were detected for the number of 120 mg/L MA reinforcers earned or the intake of this concentration prior to 2-PMPA testing (**Table 1A**).

**Table 1:** Summary of the means ± SEMs for: (A) baseline responding for MA reinforcement of the mice in Experiment 1 prior to each of their different 2-PMPA pretreatments (n=14/dose unless indicated); (B) the average responding for MA reinforcement across the 3 HEPES pretreatments of the female mice slated to receive 3, 10 or 30 mg/kg 2-PMPA pretreatment from Experiment 2 (n=11 for 3 mg/kg, n=10 for 10 and 30 mg/kg).; (C) baseline responding for MA reinforcement of the mice in Experiment 3 prior to each of their 2-PMPA pretreatments [n=11/dose for females (F); n=12/dose for males (M)]; and (D) baseline responding for sucorose reinforcement of the mice in Experiment 3 prior to either of their 2-PMPA pretreatments (n=12/dose for both males and females).

A				
Dependent Variable	2-PMPA Dose (mg/kg)			One-way ANOVA Results
Baseline	0	10	30	
***Active Hole Pokes***	73.54 ± 7.47	79.00 ± 7.16	75.56 ± 5.71	F(2,41)=0.160, p=0.853
***Inactive Hole Pokes***	19.71 ± 2.60	20.19 ± 3.06 (13)	23.20 ± 3.44	F(2,41)=0.394, p=0.677
***Response Allocation***	77.67 ± 2.72	79.92 ± 2.20 (13)	76.94 ± 1.43	F(2,41)=0.361, p=0.700
***Reinforcers Earned***	62.36 ± 6.49	66.50 ± 6.54	62.73 ± 4.23	F(2,41)=0.152, p=0.860
***MA intake (mg/kg)***	3.58 ± 0.67	3.21 ± 0.57	3.62 ± 0.52	F(2,41)=0.141, p=0.869
B				
Average	3 mg/kg	10 mg/kg	30 mg/kg	One-way ANOVA Results
***Active Hole Pokes***	36.39 ± 3.27	46.42 ± 8.33	46.42 ± 8.33	F(2,28)=0.935, p=0.405
***Inactive Hole Pokes***	9.55 ± 1.24	10.27 ± 2.37	10.27 ± 2.37	F(2,28)=0.533, p=0.593
***Response Allocation***	78.93 ± 7.20	81.91 ± 2.32	81.91 ± 2.32	F(2,28)=0.157, p=0.855
***Reinforcers Earned***	32.21 ± 2.88	40.60 ± 7.16	40.60 ± 7.16	F(2,28)=1.323, p=0.282
***MA intake (mg/kg)***	2.84 ± 0.53	3.22 ± 1.12	3.22 ± 1.12	F(2,28)=0.073, p=0.929
C				
Dependent Variable	2-PMPA Dose (mg/kg)			Sex X Dose ANOVA Results
Baseline	0	10	30	
***Active Hole Pokes***	M: 35.50 ± 7.66 F: 36.82 ± 2.90	M: 50.08 ± 7.83 F: 38.36 ± 6.03	M: 41.25 ± 5.17 F: 36.91 ± 5.16	W/I: F(2,42)<1.360, p’s>0.267 BTW: F(1,21)=0.568, p=0.459
***Inactive Hole Pokes***	M: 7.17 ± 2.35 F: 8.27 ± 1.36	M: 8.00 ± 1.41 F: 12.27 ± 3.15	M: 7.67 ± 1.89 F: 11.09 ± 2.510	W/I: F(2,42)<1.013, p’s>0.371 BTW: F(1,21)=1.571, p=0.224
***Response Allocation***	M: 85.58 ± 2.82 F: 80.66 ± 1.53	M: 87.25 ± 2.28 F: 82.09 ± 2.07	M: 82.83 ± 2.06 F: 80.76 ± 2.07	W/I: F(2,42)<1.285, p’s>0.289 BTW: F(1,21)=3.074, p=0.094
***Reinforcers Earned***	M: 33.45 ± 7.19 F: 34.20 ± 2.28	M: 42.92 ± 6.45 F: 31.55 ± 3.78	M: 35.17 ± 4.87 F: 31.27 ± 4.54	W/I: F(2,42)<1.323, p’s>0.278 BTW: F(1,21)=0.380, p=0.545
***MA intake (mg/kg)***	M: 3.28 ± 1.01 F: 2.93 ± 0.56	M: 3.81 ± .91 F: 2.68 ± 0.43	M: 3.08 ± 0.67 F: 3.11 ± 0.83	W/I: F(2,42)<0.760, p’s>0.779 BTW: F(1,21)=0.350, p=0.350
D				
Dependent Variable	2-PMPA Dose (mg/kg)		Sex X Dose ANOVA Results	
Baseline	0	100		
***Active Hole Pokes***	M: 42.50 ± 5.81 F: 30.25 ± 5.92	M: 37.25 ± 4.58 F: 30.08 ± 5.51	W/I: F(1,22)<0.579, p’s>0.455 BTW: F(1,22)=1.980, p=0.173	
***Inactive Hole Pokes***	M: 6.58 ± 1.28 F: 7.92 ± 1.36	M: 5.83 ± 0.98 F: 9.42 ± 2.01	W/I: F(1,22)<1.073, p’s>0.311 BTW: F(1,22)=1.978, p=0.174	
***Response Allocation***	M: 85.58 ± 2.39 F: 78.33 ± 1.65	M: 85.58 ± 2.39 F: 81.67 ± 2.69	W/I: F(1,22)<0.939, p’s>0.342 BTW: F(1,22)=4.780, p=0.04*	
***Reinforcers Earned***	M: 33.17 ± 5.06 F: 26.83 ± 4.79	M: 30.33 ± 3.78 F: 25.50 ± 4.58	W/I: F(1,22)<0.610, p’s>0.442 BTW: F(1,22)=0.896, p=0.354	
***Sucrose (mg/kg)***	M: 0.37 ± 0.08 F: 0.39 ± 0.05	M: 0.36 ± 0.08 F: 0.41 ± 0.07	W/I: F(1,22)<0.231, p’s>0.846 BTW: F(1,22)=0.204, p=0.656	

No significant group differences were detected for any variable in any study, indicating that mice exhibited comparable MA and/or sucrose self-administration behavior prior to 2-PMPA treatment.

For (C, D): W/I=results of within-subjects tests (Dose effect and Sex X Dose interaction); BTW=results of between-subjects tests (Sex effect).

**Figure 1:**
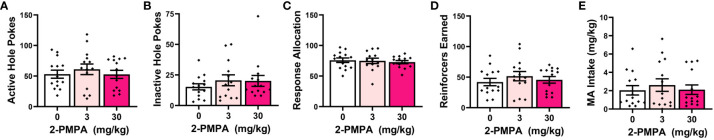
IP pretreatment with 2-PMPA does not alter the self-administration of a 120 mg/L MA solution in MA-experienced female B6 mice. Summary of the results from Experiment 1 depicting the effects of 0, 3 and 30 mg/kg 2-PMPA on: **(A)** responding in the active, MA-reinforced, hole; **(B)** responding in the inactive hole; **(C)** the allocation of total responding towards the active hole; **(D)** the number of 120 mg/L MA reinforcers earned and **(E)** MA intake (mg/kg). The data represent the means ± SEMs of 13-14 mice/dose.

As summarized in **Figure 1**, the self-administration behavior of the mice pretreated with either 3 or 30 mg/kg 2-PMPA in Experiment 1 was similar to that of the vehicle (0 mg/kg) controls. The apparent lack of any 2-PMPA effect in Experiment 1 was supported by the results of the statistical analyses, which indicated no group differences in the number of active (**Figure 1A**) [F(2,39)=0.435, p=0.651] or inactive nose-pokes (**Figure 1B**) [F(2,38)=0.581, p=0.564] and no group differences in the percentage of responses directed at the active, MA-reinforced, hole (i.e., response allocation; **Figure 1C**) [F(2,38)=0.308, p=0.737]. Further, no group differences were detected for the number of reinforcers earned (**Figure 1D**) [F(2,39)=0.324, p=0.725] or MA intake (**Figure 1E**) [F(2,39)=0.518, p=0.600] during the 1-h self-administration session. Thus, contrary to our hypothesis, 2-PMPA pretreatment did not alter the self-administration of a 120 mg/L MA solution, at least in female B6 mice with a prior history of MA-induced place-conditioning and oral self-administration.

### 3.2 Acquisition of operant-conditioning for a 200 mg/L MA solution by MA-naïve female B6 mice

**Figure 2:**
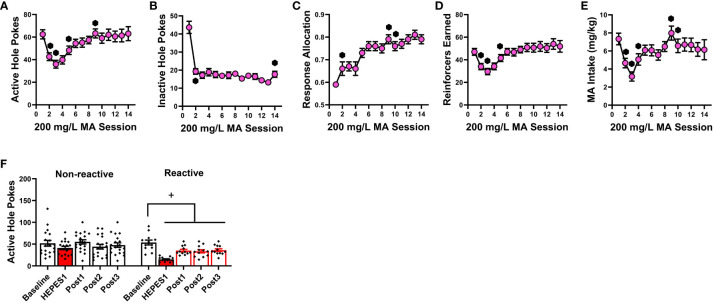
Summary of the pattern of acquisition for operant-conditioning under reinforcement by 200 mg/L MA and the influence of HEPES pretreatment on responding. Experimentally naïve female B6 mice readily acquired oral MA self-administration as indicated by: **(A)** a progressive increase in the number of nose-pokes in the “active” MA-reinforced hole and **(B)** a precipitous drop in the number of nose-pokes in the “inactive” non-reinforced hole. Correspondingly, the allocation of total responding towards the active hole progressively increased across days **(C)**, as did the number of 200 mg/L MA reinforcers earned **(D)** and MA intake **(E)**. **(F)** Despite exhibiting equivalent active hole-responding prior to initial pretreatment with 50 mM HEPES vehicle (Baseline), approximately a third of the mice in this experiment exhibited high-reactivity (Reactive) to HEPES injection as indicated by a pronounced reduction in responding following acute HEPES pretreatment (HEPES) that persisted over the 5 days following the initial pretreatment (Post1), and was apparent during the sessions following the 2^nd^ (Post2) and 3^rd^ (Post3) HEPES injection. The data represent the means ± SEMs of 14 mice. *p<0.05 vs. previous training day (t-tests); +p<0.05 vs. pre-injection baseline (paired t-tests).

Analyses of the time-courses of our measures of operant-conditioning for a 200 mg/L MA reinforcer indicated main effects of training day for the number of responses in both the active (**Figure 2A**) [F(13,364)=9.541, p<0.0001] and inactive hole (**Figure 2B**) [F(13,364)=22.317, p<0.001], as well as the allocation of total responding towards the active hole (**Figure 2C**) [F(13,364)=19.206, p<0.001]. Consistent with the temporal patterning of active hole responding (**Figure 2A**), the number of reinforcers earned during each 1-h session also varied with time (**Figure 2D**) [F(13,364)=10.294, p<0.001], as did the MA intake (**Figure 2E**) [F(13,364)=5.472, p<0.001]. Pairwise comparisons conducted across consecutive training days confirmed that responding in the active hole stabilized by the 9^th^ day of self-administration training [**Figure 2A**: for *, t(30)>2.204, p’s<0.044; for other comparisons, t(30)<1.455, p’s>0.0156]. As illustrated in **Figure 2B**, responding in the inactive hole dopped precipitously from day 1 to 2 of training and remained stable until the last day of training when a slight (but significant) uptick in responding was observed [for *, t(30)>2.963, p’s<0.007; for other comparisons, t(30)<1.625, p’s>0.0114]. Indicative of successful response acquisition, the percentage of total responses directed at the active hole (i.e., response allocation) rose progressively with training and stabilized above the 70% criterion by the 10^th^ day of training [**Figure 2C**: for *, t(30)>2.221, p<0.035; for other comparisons, t(30)<1.674, p’s<0.106]. The number of MA reinforcers stabilized early by the 5^th^ day of training [**Figure 2D**; for *, t(30)>2.243, p’s<0.033; for other comparisons, t(30)<1.89, p’s>0.06], while MA intake stabilized by the 10^th^ day of conditioning [**Figure 2E**; for *, t(30)>2.127, p’s<0.043; for other comparisons, t(30)<1.1587, p’s>0.122]. The data in **Figure 2** demonstrate that 200 mg/L MA is reinforcing in drug-naïve female B6 mice and can effectively entice high levels of MA-appropriate responding with intakes averaging around 6 mg/kg during a 1-h period.

### 3.3 2-PMPA effects on operant-responding for 200 mg/L MA by female B6 mice

Having established the reinforcing properties of 200 mg/L MA (**Figure 2**), we then tested the effects of 2-PMPA (3, 10 and 30 mg/kg, IP) on our measures of MA reinforcement. Unlike Experiment 1, the mice in this study had no prior experience with i.p. injection procedures. Thus, the mice in Experiment 2 were first pretreated with HEPES vehicle upon the stabilization of MA self-administration behavior to habituate the mice to the injection procedures. Visual inspection of the data for active nose-poke responding following this initial HEPES injection suggested that the injection reduced responding to less than 45% of baseline in 12 out of the 31 mice and the responding in these “reactive” mice did not recover back to pre-injection baseline over the course of the next 5 days following injection or during the intervals between the two additional HEPES injections. This observation was supported by the results of a within-subjects ANOVA conducted on the number of active nose-pokes across these different time-points between “reactive” and “non-reactive” mice [Reactivity effect: F(1,29)=7.005, p=0.013; Session: F(4,116)=9.820, p<0.001; Reactivity X Session: F(4,116)=3.391, p=0.012]. As illustrated in **Figure 2F** (left), the initial HEPES pretreatment did not significantly affect active hole-responding in the “non-reactive” mice, relative to their pre-injection baseline [t(11)=1.805, p=0.088] and responding recovered to pre-injection baseline levels following each HEPES injection as indicated by no difference between the pre-injection baseline responding and responding following their first, second or third HEPES injection (paired t-tests, t’s<1.996, p’s>0.060). In contrast, the initial HEPES injection resulted in a marked reduction in active hole-responding in the “reactive” mice (**Figure 2F**, right) [t(11)=7.830, p<0.001] and the responding of “reactive” mice remained significantly lower than pre-injection baseline levels during each post-injection period [for post-injection 1, t(11)=4.077, p=0.002; for post-injection 2, t(11)=2.519, p=0.029; for post-injection 3, t(11)=2.382, p=0.036]. Thus, careful attention was made to equate the average responding under HEPES across the three 2-PMPA doses and a lack of group differences in behavior under HEPES was confirmed by one-way ANOVAs for all variables (see **Table 1B**).

**Figure 3:**
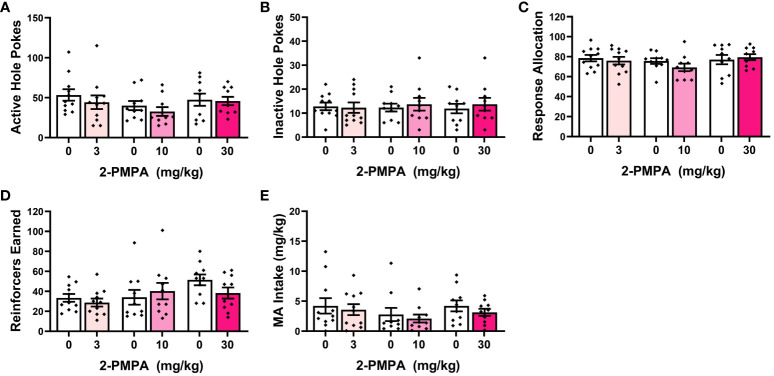
IP pretreatment with 2-PMPA does not alter the self-administration of a 200 mg/L MA solution in previously MA-naïve female B6 mice. Summary of the results from Experiment 2 depicting the effects of 3, 10 and 30 mg/kg 2-PMPA on: **(A)** responding in the active, MA-reinforced, hole; **(B)** responding in the inactive hole; **(C)** the allocation of total responding towards the active hole; **(D)** the number of 200 mg/L MA reinforcers earned and **(E)** MA intake (mg/kg). The data represent the means ± SEMs of 9-11 mice/dose.

Out of concern that differential responding to the HEPES injections between “reactive” versus “non-reactive” mice might influence the magnitude of any 2-PMPA effects, Phenotype was included as a between-subjects factor upon initial data analyses in Experiment 2. As provided in the **Supplementary Results**, the results of the Phenotype X Dose X Pretreatment ANOVAs indicated no group differences or interactions with the Phenotype factor and thus, the data were collapsed across the “reactive” and “non-reactive” mice for final analyses. As illustrated in **Figure 3**, none of the 2-PMPA doses significantly altered our measures of MA reinforcement, relative to the effects of HEPES pretreatment [Pretreatment X Dose ANOVA’s: for active hole responding, F(1,28)<2.864, p’s>0.101 and F(2,28)=0.401, p=0.67; for inactive hole responding, F(1,28)<0.327, p’s>0.571 and F(2,28)=0.225, p=0.800; for response allocation, F(1,28)<1.138, p’s>0.294 and F(2,28)=1.507, p=0.239; for reinforcers earned, F(1,28)<1.831, p’s>0.186 and F(2,28)=0.203, p=0.817; and for MA intake, F(1,28)<1.987, p’s>0.169 and F(2,28)=0.062, p=0.940]. Thus, systemic pretreatment with 2-PMPA also failed to alter the self-administration of 200 mg/L MA in female B6 mice with no prior MA history.

### 3.4 Higher dose 2-PMPA effects on operant-responding for 200 mg/L MA by female versus male B6 mice

Experiment 3 examined whether the negative results from Experiments 1 and 2 might reflect insufficient 2-PMPA dosing or the employ of female subjects by comparing effects of 30 and 100 mg/kg 2-PMPA between male and female mice. One female was dropped from the MA phase of the study prior to 2-PMPA pretreatment for failing to meet the acquisition criteria after 2 weeks of self-administration training. A summary of the baseline behavior prior to each 2-PMPA dose during the MA self-administration phase of Experiment 3 is presented **Table 1C**. No group differences were detected for any baseline measure prior to each of the three pretreatment days.

**Figure 4:**
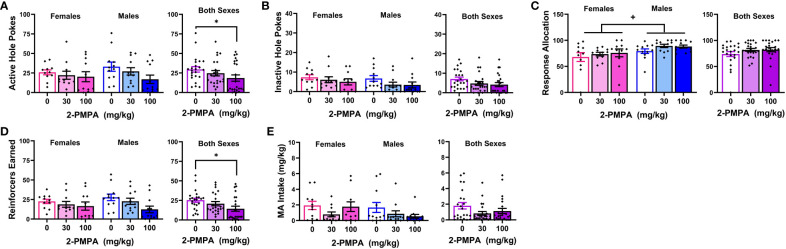
IP pretreatment with 2-PMPA alters some measures of 200 mg/L MA reinforcement at the 100 mg/kg dose. Summary of the results from the MA phase of Experiment 3 depicting the effects of 0, 30 and 100 mg/kg 2-PMPA on: **(A)** responding in the active, MA-reinforced, hole; **(B)** responding in the inactive hole; **(C)** the allocation of total responding towards the active hole; **(D)** the number of 120 mg/L MA reinforcers earned and **(E)** MA intake (mg/kg) by female (left) and male (right) mice. The data represent the means ± SEMs of 11 females and 12 males. *p<0.05 vs. 0 mg/kg 2-PMPA (Dose effect; paired t-tests); +p<0.05 vs. females (Sex effect).

**Figure 4** presents the data for the effects of 30 and 100 mg/kg 2-PMPA on our measures of MA reinforcement in female and male mice. With the exception of higher MA-appropriate response allocation in males versus females (**Figure 4C**) [Sex effect: F(1,21)=4.885, p=0.038], Sex X Dose ANOVAs conducted on the data for Experiment 3 failed to indicate any significant Sex effects or interactions [Sex effects, F(1,21)<2.094, p’s>0.162; Sex X Dose interactions, F(2,42)<1.403, p’s>0.257). Thus, the data are also presented as collapsed across sex to better visualize main Dose effects. As illustrated in **Figure 4A**, 100 mg/kg 2-PMPA reduced responding in the active hole, while the 30 mg/kg was again without effect [Dose effect: F(2,42)=3.384, p=0.043; paired t-tests: for 0 vs. 30, t(22)=1.695, p=0.104; for 0 vs. 100: t(22)=2.319, p=0.030]. In contrast, neither dose significantly altered responding in the inactive hole, (**Figure 4B**) [Dose effect: F(2,42)=0.707, p=0.499] nor did they significantly affect response allocation, although a dose-dependent trend for increased response allocation towards the MA-reinforced hole was noted (**Figure 4C**) [Dose effect: F(2,42)=2.700, p=0.079]. Consistent with the data for active hole-responding, the 100 mg/kg 2-PMPA dose significantly lowered the number of MA reinforcers earned (**Figure 4D**) [Dose effect: F(2,42)=5.275, p=0.009; paired t-tests: 0 vs. 30, t(22)=1.542, p=0.137; for 0 vs. 100: t(22)=2.939, p=0.008]. Although MA intake trended downward with 2-PMPA pretreatment, neither dose significantly lower the amount of MA consumed likely owing the relatively high variability in intake under all three doses (**Figure 4E**) [Dose effect: F(2,42)=2.587, p=0.087]. These results from Experiment 3 support insufficient dosing, rather than the sex of the subjects, as a procedural factor impacting the ability to detect an effect of 2-PMPA on MA reinforcement as 100 mg/kg 2-PMPA effectively lowered some measures of MA reinforcement in both male and female mice.

**Figure 5:**
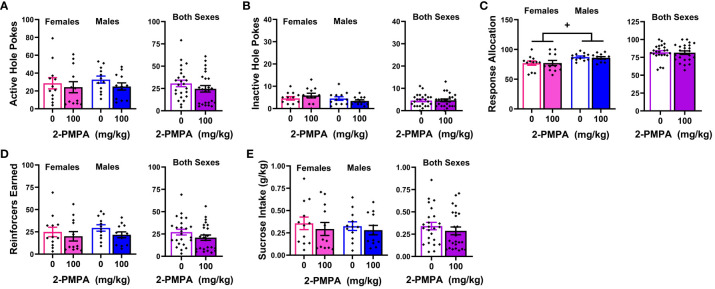
IP pretreatment with 2-PMPA does not alter the self-administration of a 20% sucrose solution by male or female B6 mice. Summary of the results from the sucrose phase of Experiment 3 depicting the effects of 0 versus 100 mg/kg 2-PMPA on: **(A)** responding in the active, sucrose-reinforced, hole; **(B)** responding in the inactive hole; **(C)** the allocation of total responding towards the active hole; **(D)** the number of 20% sucrose reinforcers earned and **(E)** sucrose intake (g/kg) by female (left) and male (right) mice. The data represent the means ± SEMs of 12 mice/sex. +p<0.05 vs. females (Sex effect).

A summary of the baseline behavior prior to each of the two 2-PMPA doses during the sucrose self-administration phase of Experiment 3 is presented **Table 1D**. With the exception of higher response allocation towards the active hole in males versus female mice, no group differences were detected for any baseline measure prior to either pretreatment day. We also detected higher response allocation by males versus females during the session prior to which mice received pretreatment (**Figure 5C**) [Sex effect: F(1,22)=5.820, p=0.025], but this variable was the only to exhibit a sex difference [Sex effect for other variables, F(1,22)<2.082, p’s>0.162]. In contrast to our results for MA reinforcement, we failed to detect any statistically significant Dose effects or interactions for any of the variables in the study of the effects of 100 mg/kg 2-PMPA on sucrose reinforcement (**Figure 5**) [Dose effect and interactions: for active hole, F(1,22)<2.600, p’s>0.120; for inactive hole, F(1,22)<3.404, p’s>0.078; for response allocation: F(1,22)<0.183, p’s>0.673; for reinforcers earned, F(1,22)<4.009, p’s>0.059; for sucrose intake (mg/kg), F(1,22)<1.081, p’s>0.309]. The only variable to exhibit a trend for a Dose effect was the number of sucrose reinforcers earned, which tended to be lower in mice treated with 100 mg/kg 2-PMPA (**Figure 1D**) [F(1,22)=4.008, p=0.058]. These results from Experiment 3 argue against large off-target effects of the 100 mg/kg 2-PMPA dose (e.g., deficits in motor, cognitive, or reward processing) as mechanisms through which this dose affects certain indices of MA reinforcement.

## 4 Discussion

The NAALADase inhibitor 2-PMPA is reported to be effective at reducing the reinforcing and/or rewarding properties of both cocaine (46, 50, 51) and MDPV (52) in laboratory rodents. Thus, we conducted three small studies to determine whether the putative “anti-addictive” effects of 2-PMPA might extend to MA, using a procedurally facile model of oral MA self-administration in B6 mice. Despite employing a comparable pretreatment interval (30 min) and route of administration (IP) as those in earlier cocaine self-administration studies (46, 51), we failed to detect any effect of 30 mg/kg 2-PMPA pretreatment on oral MA self-administration behavior in any of our experiments. However, the 100 mg/kg 2-PMPA dose significantly reduced responding in the MA-reinforced active hole, as well as the number of reinforcers earned, but did not affect MA intake or lower response allocation. Below, we discuss our findings within the context of our current knowledge regarding the effects of 2-PMPA on substance use disorder-related behavior.

The failure of 30 mg/kg 2-PMPA to alter MA self-administration in our study does not likely reflect a floor or ceiling effect on behavior as the mice in all three experiments exhibited comparably high levels of MA-appropriate responding during baseline (**Tables 1A–C**). The mice in Experiments 1 and 2 also exhibited high levels of MA-appropriate responding during testing (~50 nose-pokes in 1 h; **Figures 1A** and **3A**), while the mice in Experiment 3 exhibited lower responding during testing (~25 nose-pokes; **Figure 4A**). Further, MA intakes averaged approximately 2, 6 and 3 mg/kg during a 1-h session, respectively in Experiments 1, 2 and 3 (**Table 1**, **Figures 1E**, **2E** and **4E**). To the best of our knowledge, this study is the first to examine the effects of 2-PMPA upon indices of stimulant reinforcement in mice and we started our investigation with doses reported to be sufficient in mouse to reduce [^3^H]-NAAG hydrolysis in brain by 50% [10 mg/kg; (58)] and to exert pro-cognitive effects [0.2-10 mg/kg; (52)]. Given the reported low bioavailability of 2-PMPA when injected IP [(59, 60); but see (52)], it is possible that the 30 mg/kg 2-PMPA dose was insufficient to elicit a behavioral effect in MA-experienced mice. Indeed, most earlier studies that examined the behavioral effects of 2-PMPA in mice employed doses of 50-100 mg/kg (IP) [e.g., (39, 48–50, 52, 58–62)]. Interestingly, the results of these prior mouse studies indicated little to no effect of acute pretreatment with these higher 2-PMPA doses on the behavior of cocaine-treated mice (48, 52), while acute pretreatment with 100 and 150 mg/kg 2-PMPA produced equivocally reduced the locomotor stimulatory effects of acute amphetamine (58). Here, pretreatment with the 100 mg/kg 2-PMPA dose lowered some aspects of MA-reinforced responding in B6 mice (**Figures 4A, D**), in a manner consistent with prior reports for IV stimulant reinforcement in rats (46, 49–51). These results suggest that mice may require higher 2-PMPA doses when this inhibitor is administered acutely to observe effects on stimulant-induced changes in behavior. Supporting this notion, 100 mg/kg 2-PMPA, while sufficient to lower MA-reinforced responding and the number of reinforcers earned, did not alter MA intake (**Figure 4E**). Alternatively, 100 mg/kg 2-PMPA may be more effective at interrupting stimulant-induced behavior in mice with repeated treatment, based on the results of cocaine-induced locomotor sensitization and kindling studies (49, 50). Whether a more complete 2-PMPA effect on MA self-administration and/or MA-induced changes in locomotor behavior in mice would be observed at higher doses or with repeated treatment remains to be determined.

Interestingly, while 100 mg/kg 2-PMPA (IP) is required to block the locomotor stimulatory effect of amphetamine when administered acutely, a 2-PMPA effect on phencyclidine-induced locomotion is apparent at a 10-folder lower dose (52). The differences in the potency of 2-PMPA for reducing PCP- versus amphetamine-induced hyperlocomotion raises the possibility that 2-PMPA may be more effective at restoring drug-induced perturbations in glutamate (which is directly targeted by PCP) than dopamine (which is elevated by amphetamine). However, arguing against this suggestion are the results of an *in vivo* microdialysis study demonstrating that 2-PMPA is equipotent at reducing extracellular dopamine and glutamate levels within the nucleus accumbens, with a 40-50% reduction in baseline neurotransmitter levels observed when rats were injected IP with either 30 or 100 mg/kg 2-PMPA. Furthermore, both 2-PMPA doses blunted the capacity of cocaine to elevate levels of both dopamine and glutamate in the nucleus accumbens. However, in contrast to 2-PMPA’s effect on basal neurotransmitter levels, the effects of 2-PMPA on cocaine-stimulated neurotransmitter release was dose-dependent (46). Whether 2-PMPA alters basal dopamine and glutamate levels in mice and whether it can also blunt the capacity of MA to elevate dopamine and glutamate in any brain region is currently not known. Given that certain neuroadaptations within the corticoaccumbens glutamate pathways (including receptor and transporter expression) induced by repeated MA exposure are distinguishable from those induced by cocaine [e.g., (19, 34)], it would not be surprising if 2-PMPA affects MA-induced changes in glutamate, (and perhaps dopamine or other neurotransmitter systems) in a manner distinct from its interactions with cocaine-induced changes in neurotransmitter release to possibility account for its more robust effects on cocaine versus MA self-administration behavior.

An alternate explanation for our results might relate to the route of reinforcer administration given that mice orally consumed MA in the present study, while prior studies demonstrating robust 2-PMPA efficacy employed an IV route of reinforcer administration (46, 51). While no study has yet assayed for the effect of 2-PMPA on the IV self-administration of any other drug of abuse besides cocaine, IP pretreatment with 2-PMPA (50 and 100 mg/kg, IP) reduces alcohol drinking in female alcohol-preferring P rats when assessed under short-access (1 h) home cage drinking procedures (57). Such results align with the present findings for 100 mg/kg 2-PMPA in female mice (**Figure 4**), demonstrating that 100 mg/kg 2-PMPA (IP) is effective at reducing oral drug intake in both female rats and mice. While most studies concerning the behavioral effects of 2-PMPA have been conducted in male rodents, our results from Experiment 3, in addition to the study of McKinzie et al. (57) and others [e.g., (63–66)] indicate clearly that IP administration of 2-PMPA effectively influences behavior and pathophysiology in female rodents. Thus, the likelihood that our negative results at 3-30 mg/kg 2-PMPA relate to the sex of our subjects is low. Indeed, under the current oral MA self-administration procedures, the 30 mg/kg dose was similarly ineffective, while the 100 mg/kg dose was equally effective, in male and female mice (**Figure 4**). However, it should be noted that in the alcohol-drinking study of McKinzie et al. (57), the highest 2-PMPA tested (200 mg/kg) had no effect on alcohol intake, while both 50 and 100 mg/kg 2-PMPA reduced alcohol intake by 25%. Thus, the dose-response function for 2-PMPA’s effects on alcohol-drinking is inverted U-shaped. Admittedly, we did not assay for 2-PMPA effects at doses higher than 100 mg/kg herein. However, according to this alcohol study (57), the 2-PMPA doses employed herein (3-100 mg/kg) lie on the ascending limb of the inverted U-shaped dose-response function and thus, our negative results under 30 mg/kg and partial effects under 100 mg/kg 2-PMPA do not likely reflect over-dosing.

Our negative results might relate to the MA concentrations employed as the reinforcer. This possibility was raised based on the study by Xi et al. (46), in which the effects of 2-PMPA upon IV cocaine self-administration varied considerably as a function of the dose of the IV cocaine reinforcer. More specifically, 2-PMPA pretreatment (10-100 mg/kg, IP) did not alter cocaine self-administration when 0.50 mg/kg cocaine/infusion served as the reinforcer – a finding that was replicated within their report. However, when the cocaine reinforcer dose was lowered, both 10 and 30 mg/kg 2-PMPA effectively reduced the self-administration of 0.06 and 0.12 mg/kg/infusion cocaine, while only 30 mg/kg 2-PMPA lowered the self-administration of 0.25 mg/kg/infusion cocaine (46). As the goal of our pilot studies was to characterize the dose-response function of 2-PMPA on MA reinforcement and not how 2-PMPA alters the dose-response function for MA, it remains to be determined whether our negative results might relate to our selection of MA reinforcer concentrations. Thus, while the results of our studies argue against any effect of the 30 mg/kg 2-PMPA dose on MA reinforcement, it would be important in future studies to determine whether the present results might relate to an interaction between the dose of 2-PMPA administered and the concentration of the MA reinforcer employed.

Finally, it is worth highlighting that in contrast to its effects on some measures of MA reinforcement (**Figure 4**), 100 mg/kg 2-PMPA did not significantly impact any measure of sucrose reinforcement in Experiment 3 (**Figure 5**). Our negative results for oral sucrose self-administration by mice are consistent with earlier sucrose studies in rat (46, 51, 57) indicating that the capacity of this NAADALase inhibitor to reduce the reinforcing/rewarding properties of across different drugs of abuse does not generalize to non-drug reinforcers. Further, these negative results are consistent with evidence from locomotor studies in both rats and mice indicating no effect of 2-PMPA pretreatment, at least at the doses employed herein, on spontaneous locomotor activity (49, 50, 52, 58). Thus, the “anti-addiction” efficacy of 2-PMPA cannot be readily explained by off-target effects on general cognitive, reward or motor processing of relevance to its potential side effect profile as a therapeutic.

### 4.1 Conclusions

Systemic pretreatment with the NAALADase inhibitor 2-PMPA, administered at doses demonstrated to be effective at reducing IV cocaine self-administration, reduced some signs of oral MA reinforcement in mice without altering MA intake. Our findings align with the potential therapeutic efficacy of 2-PMPA for treating substance use disorder, although more research is required to determine how factors such as 2-PMPA dose, chronicity of 2-PMPA treatment and MA reinforcer concentration influence its potential “anti-addictive” effects.

## Data availability statement

The raw data supporting the conclusions of this article will be made available by the authors, without undue reservation.

## Ethics statement

The animal study was approved by Institutional Animal Care and Use Committee of the University of California, Santa Barbara. The study was conducted in accordance with the local legislation and institutional requirements.

## Author contributions

EF: Conceptualization, Funding acquisition, Methodology, Writing – review & editing, Data curation, Investigation. AN: Writing – review & editing, Data curation, Investigation. JC: Writing – review & editing, Data curation, Investigation. JL: Writing – review & editing, Data curation, Investigation. KS: Conceptualization, Funding acquisition, Methodology, Writing – review & editing, Formal analysis, Project administration, Supervision, Visualization, Writing – original draft.

## Supplementary material

Supplemental Results To determine whether the “reactive” phenotype exhibited by a subset of the female mice in Experiment 2 when injected with HEPEs influenced their behavior under the influence of 2-PMPA, the data were initially analyzed using Phenotype X Dose X Pretreatment ANOVAs. As summarized in the SPSS Output files from this analysis, we detected no group differences or interactions with the Phenotype factor for any of the variables investigated. Thus, the data were collapsed across the “reactive” and “non-reactive” mice for final analyses, which are presented in the main text.
