# Supplementary material for: Effects of systemic pretreatment with the NAALADase inhibitor 2-PMPA on oral methamphetamine reinforcement in C57BL/6J mice
Source: Front Psychiatry. 2024 Apr 4;15:1297275. doi: 10.3389/fpsyt.2024.1297275 (PMC11024460; doi:10.3389/fpsyt.2024.1297275)
Supplement: Supplemental Results — To determine whether the “reactive” phenotype exhibited by a subset of the female mice in Experiment 2 when injected with HEPEs influenced their behavior under the influence of 2-PMPA, the data were initially analyzed using Phenotype X Dose X Pretreatment ANOVAs. As summarized in the SPSS Output files from this analysis, we detected no group differences or interactions with the Phenotype factor for any of the variables investigated. Thus, the data were collapsed across the “reactive” and “non-reactive” mice for final analyses, which are presented in the main text. [file DataSheet_1.pdf]

## Supplemental Results

To determine whether the "reactive" phenotype exhibited by a subset of the female mice in Experiment 2 when injected with HEPEs influenced their behavior under the influence of 2-PMPA, the data were initially analyzed using Phenotype X Dose X Pretreatment ANOVAs. As summarized in the SPSS Output files from this analysis, we detected no group differences or interactions with the Phenotype factor for any of the variables investigated. Thus, the data were collapsed across the "reactive" and "non-reactive" mice for final analyses, which are presented in the main text.

Phenotype X Dose X Pretreatment ANOVA
Active Hole Pokes

**Tests of Within-Subjects Effects**

Measure: MEASURE_1

| Source | | Type III Sum of Squares | df | Mean Square | F | Sig. |
|---|---|---|---|---|---|---|
| PreRx | Sphericity Assumed | 378.361 | 1 | 378.361 | 1.775 | .195 |
| | Greenhouse-Geisser | 378.361 | 1.000 | 378.361 | 1.775 | .195 |
| | Huynh-Feldt | 378.361 | 1.000 | 378.361 | 1.775 | .195 |
| | Lower-bound | 378.361 | 1.000 | 378.361 | 1.775 | .195 |
| PreRx * Dosemgkg | Sphericity Assumed | 111.159 | 2 | 55.579 | .261 | .773 |
| | Greenhouse-Geisser | 111.159 | 2.000 | 55.579 | .261 | .773 |
| | Huynh-Feldt | 111.159 | 2.000 | 55.579 | .261 | .773 |
| | Lower-bound | 111.159 | 2.000 | 55.579 | .261 | .773 |
| PreRx * Pheno | Sphericity Assumed | 16.329 | 1 | 16.329 | .077 | .784 |
| | Greenhouse-Geisser | 16.329 | 1.000 | 16.329 | .077 | .784 |
| | Huynh-Feldt | 16.329 | 1.000 | 16.329 | .077 | .784 |
| | Lower-bound | 16.329 | 1.000 | 16.329 | .077 | .784 |
| PreRx * Dosemgkg * Pheno | Sphericity Assumed | 104.202 | 2 | 52.101 | .244 | .785 |
| | Greenhouse-Geisser | 104.202 | 2.000 | 52.101 | .244 | .785 |
| | Huynh-Feldt | 104.202 | 2.000 | 52.101 | .244 | .785 |
| | Lower-bound | 104.202 | 2.000 | 52.101 | .244 | .785 |
| Error(PreRx) | Sphericity Assumed | 5328.762 | 25 | 213.150 | | |
| | Greenhouse-Geisser | 5328.762 | 25.000 | 213.150 | | |
| | Huynh-Feldt | 5328.762 | 25.000 | 213.150 | | |
| | Lower-bound | 5328.762 | 25.000 | 213.150 | | |

**Tests of Between-Subjects Effects**

Measure: MEASURE_1
Transformed Variable: Average

| Source | Type III Sum of Squares | df | Mean Square | F | Sig. |
|---|---|---|---|---|---|
| Intercept | 97804.921 | 1 | 97804.921 | 141.160 | <.001 |
| Dosemgkg | 2578.045 | 2 | 1289.022 | 1.860 | .177 |
| Pheno | 941.481 | 1 | 941.481 | 1.359 | .255 |
| Dosemgkg * Pheno | 3167.650 | 2 | 1583.825 | 2.286 | .123 |
| Error | 17321.679 | 25 | 692.867 | | |

Phenotype X Dose X Pretreatment ANOVA
Inactive Hole Pokes

**Tests of Within-Subjects Effects**

Measure: MEASURE_1

| Source | | Type III Sum of Squares | df | Mean Square | F | Sig. |
|---|---|---|---|---|---|---|
| PreRx | Sphericity Assumed | 41.261 | 1 | 41.261 | 1.325 | .261 |
| | Greenhouse-Geisser | 41.261 | 1.000 | 41.261 | 1.325 | .261 |
| | Huynh-Feldt | 41.261 | 1.000 | 41.261 | 1.325 | .261 |
| | Lower-bound | 41.261 | 1.000 | 41.261 | 1.325 | .261 |
| PreRx * Dosemgkg | Sphericity Assumed | 71.593 | 2 | 35.796 | 1.150 | .333 |
| | Greenhouse-Geisser | 71.593 | 2.000 | 35.796 | 1.150 | .333 |
| | Huynh-Feldt | 71.593 | 2.000 | 35.796 | 1.150 | .333 |
| | Lower-bound | 71.593 | 2.000 | 35.796 | 1.150 | .333 |
| PreRx * Pheno | Sphericity Assumed | 54.242 | 1 | 54.242 | 1.742 | .199 |
| | Greenhouse-Geisser | 54.242 | 1.000 | 54.242 | 1.742 | .199 |
| | Huynh-Feldt | 54.242 | 1.000 | 54.242 | 1.742 | .199 |
| | Lower-bound | 54.242 | 1.000 | 54.242 | 1.742 | .199 |
| PreRx * Dosemgkg * Pheno | Sphericity Assumed | 215.434 | 2 | 107.717 | 3.459 | .047 |
| | Greenhouse-Geisser | 215.434 | 2.000 | 107.717 | 3.459 | .047 |
| | Huynh-Feldt | 215.434 | 2.000 | 107.717 | 3.459 | .047 |
| | Lower-bound | 215.434 | 2.000 | 107.717 | 3.459 | .047 |
| Error(PreRx) | Sphericity Assumed | 778.512 | 25 | 31.140 | | |
| | Greenhouse-Geisser | 778.512 | 25.000 | 31.140 | | |
| | Huynh-Feldt | 778.512 | 25.000 | 31.140 | | |
| | Lower-bound | 778.512 | 25.000 | 31.140 | | |

**Tests of Between-Subjects Effects**

Measure: MEASURE_1
Transformed Variable: Average

| Source | Type III Sum of Squares | df | Mean Square | F | Sig. |
|---|---|---|---|---|---|
| Intercept | 9129.517 | 1 | 9129.517 | 160.033 | <.001 |
| Dosemgkg | 13.663 | 2 | 6.831 | .120 | .888 |
| Pheno | 11.585 | 1 | 11.585 | .203 | .656 |
| Dosemgkg * Pheno | 158.090 | 2 | 79.045 | 1.386 | .269 |
| Error | 1426.190 | 25 | 57.048 | | |

Phenotype X Dose X Pretreatment ANOVA
Response Allocation

**Tests of Within-Subjects Effects**

Measure: MEASURE_1

| Source | | Type III Sum of Squares | df | Mean Square | F | Sig. |
|---|---|---|---|---|---|---|
| PreRx | Sphericity Assumed | 72.233 | 1 | 72.233 | 1.033 | .319 |
| | Greenhouse-Geisser | 72.233 | 1.000 | 72.233 | 1.033 | .319 |
| | Huynh-Feldt | 72.233 | 1.000 | 72.233 | 1.033 | .319 |
| | Lower-bound | 72.233 | 1.000 | 72.233 | 1.033 | .319 |
| PreRx * Dosemgkg | Sphericity Assumed | 144.645 | 2 | 72.323 | 1.035 | .370 |
| | Greenhouse-Geisser | 144.645 | 2.000 | 72.323 | 1.035 | .370 |
| | Huynh-Feldt | 144.645 | 2.000 | 72.323 | 1.035 | .370 |
| | Lower-bound | 144.645 | 2.000 | 72.323 | 1.035 | .370 |
| PreRx * Pheno | Sphericity Assumed | 5.914 | 1 | 5.914 | .085 | .774 |
| | Greenhouse-Geisser | 5.914 | 1.000 | 5.914 | .085 | .774 |
| | Huynh-Feldt | 5.914 | 1.000 | 5.914 | .085 | .774 |
| | Lower-bound | 5.914 | 1.000 | 5.914 | .085 | .774 |
| PreRx * Dosemgkg * Pheno | Sphericity Assumed | 46.194 | 2 | 23.097 | .330 | .722 |
| | Greenhouse-Geisser | 46.194 | 2.000 | 23.097 | .330 | .722 |
| | Huynh-Feldt | 46.194 | 2.000 | 23.097 | .330 | .722 |
| | Lower-bound | 46.194 | 2.000 | 23.097 | .330 | .722 |
| Error(PreRx) | Sphericity Assumed | 1747.313 | 25 | 69.893 | | |
| | Greenhouse-Geisser | 1747.313 | 25.000 | 69.893 | | |
| | Huynh-Feldt | 1747.313 | 25.000 | 69.893 | | |
| | Lower-bound | 1747.313 | 25.000 | 69.893 | | |

**Tests of Between-Subjects Effects**

Measure: MEASURE_1
Transformed Variable: Average

| Source | Type III Sum of Squares | df | Mean Square | F | Sig. |
|---|---|---|---|---|---|
| Intercept | 304566.770 | 1 | 304566.770 | 1344.098 | <.001 |
| Dosemgkg | 303.299 | 2 | 151.649 | .669 | .521 |
| Pheno | 60.975 | 1 | 60.975 | .269 | .609 |
| Dosemgkg * Pheno | 53.831 | 2 | 26.916 | .119 | .888 |
| Error | 5664.891 | 25 | 226.596 | | |

Phenotype X Dose X Pretreatment ANOVA
Reinforcers Earned

**Tests of Within-Subjects Effects**

Measure: MEASURE_1

| Source | | Type III Sum of Squares | df | Mean Square | F | Sig. |
|---|---|---|---|---|---|---|
| PreRx | Sphericity Assumed | 149.386 | 1 | 149.386 | 1.082 | .308 |
| | Greenhouse-Geisser | 149.386 | 1.000 | 149.386 | 1.082 | .308 |
| | Huynh-Feldt | 149.386 | 1.000 | 149.386 | 1.082 | .308 |
| | Lower-bound | 149.386 | 1.000 | 149.386 | 1.082 | .308 |
| PreRx * Dosemgkg | Sphericity Assumed | 24.277 | 2 | 12.138 | .088 | .916 |
| | Greenhouse-Geisser | 24.277 | 2.000 | 12.138 | .088 | .916 |
| | Huynh-Feldt | 24.277 | 2.000 | 12.138 | .088 | .916 |
| | Lower-bound | 24.277 | 2.000 | 12.138 | .088 | .916 |
| PreRx * Pheno | Sphericity Assumed | 8.381 | 1 | 8.381 | .061 | .807 |
| | Greenhouse-Geisser | 8.381 | 1.000 | 8.381 | .061 | .807 |
| | Huynh-Feldt | 8.381 | 1.000 | 8.381 | .061 | .807 |
| | Lower-bound | 8.381 | 1.000 | 8.381 | .061 | .807 |
| PreRx * Dosemgkg * Pheno | Sphericity Assumed | 87.037 | 2 | 43.519 | .315 | .732 |
| | Greenhouse-Geisser | 87.037 | 2.000 | 43.519 | .315 | .732 |
| | Huynh-Feldt | 87.037 | 2.000 | 43.519 | .315 | .732 |
| | Lower-bound | 87.037 | 2.000 | 43.519 | .315 | .732 |
| Error(PreRx) | Sphericity Assumed | 3451.175 | 25 | 138.047 | | |
| | Greenhouse-Geisser | 3451.175 | 25.000 | 138.047 | | |
| | Huynh-Feldt | 3451.175 | 25.000 | 138.047 | | |
| | Lower-bound | 3451.175 | 25.000 | 138.047 | | |

**Tests of Between-Subjects Effects**

Measure: MEASURE_1
Transformed Variable: Average

| Source | Type III Sum of Squares | df | Mean Square | F | Sig. |
|---|---|---|---|---|---|
| Intercept | 70920.321 | 1 | 70920.321 | 125.006 | <.001 |
| Dosemgkg | 2423.672 | 2 | 1211.836 | 2.136 | .139 |
| Pheno | 625.571 | 1 | 625.571 | 1.103 | .304 |
| Dosemgkg * Pheno | 2540.227 | 2 | 1270.114 | 2.239 | .128 |
| Error | 14183.425 | 25 | 567.337 | | |

Phenotype X Dose X Pretreatment ANOVA
MA intake (mg/kg)

**Tests of Within-Subjects Effects**

Measure: MEASURE_1

| Source | | Type III Sum of Squares | df | Mean Square | F | Sig. |
|---|---|---|---|---|---|---|
| PreRx | Sphericity Assumed | 4.932 | 1 | 4.932 | .928 | .345 |
| | Greenhouse-Geisser | 4.932 | 1.000 | 4.932 | .928 | .345 |
| | Huynh-Feldt | 4.932 | 1.000 | 4.932 | .928 | .345 |
| | Lower-bound | 4.932 | 1.000 | 4.932 | .928 | .345 |
| PreRx * Dosemgkg | Sphericity Assumed | .003 | 2 | .001 | .000 | 1.000 |
| | Greenhouse-Geisser | .003 | 2.000 | .001 | .000 | 1.000 |
| | Huynh-Feldt | .003 | 2.000 | .001 | .000 | 1.000 |
| | Lower-bound | .003 | 2.000 | .001 | .000 | 1.000 |
| PreRx * Pheno | Sphericity Assumed | 3.675 | 1 | 3.675 | .692 | .413 |
| | Greenhouse-Geisser | 3.675 | 1.000 | 3.675 | .692 | .413 |
| | Huynh-Feldt | 3.675 | 1.000 | 3.675 | .692 | .413 |
| | Lower-bound | 3.675 | 1.000 | 3.675 | .692 | .413 |
| PreRx * Dosemgkg * Pheno | Sphericity Assumed | 2.503 | 2 | 1.252 | .236 | .792 |
| | Greenhouse-Geisser | 2.503 | 2.000 | 1.252 | .236 | .792 |
| | Huynh-Feldt | 2.503 | 2.000 | 1.252 | .236 | .792 |
| | Lower-bound | 2.503 | 2.000 | 1.252 | .236 | .792 |
| Error(PreRx) | Sphericity Assumed | 132.812 | 25 | 5.312 | | |
| | Greenhouse-Geisser | 132.812 | 25.000 | 5.312 | | |
| | Huynh-Feldt | 132.812 | 25.000 | 5.312 | | |
| | Lower-bound | 132.812 | 25.000 | 5.312 | | |

**Tests of Between-Subjects Effects**

Measure: MEASURE_1
Transformed Variable: Average

| Source | Type III Sum of Squares | df | Mean Square | F | Sig. |
|---|---|---|---|---|---|
| Intercept | 627.879 | 1 | 627.879 | 48.219 | <.001 |
| Dosemgkg | 50.659 | 2 | 25.330 | 1.945 | .164 |
| Pheno | .021 | 1 | .021 | .002 | .968 |
| Dosemgkg * Pheno | 62.999 | 2 | 31.499 | 2.419 | .110 |
| Error | 325.537 | 25 | 13.021 | | |
